# A brief patient-reported outcome instrument for primary care: German translation and validation of the Measure Yourself Medical Outcome Profile (MYMOP)

**DOI:** 10.1186/s12955-014-0112-5

**Published:** 2014-07-19

**Authors:** Katja Hermann, Katharina Kraus, Kathrin Herrmann, Stefanie Joos

**Affiliations:** Department of General Practice and Health Services Research, University Hospital Heidelberg, Vossstr. 2, Heidelberg, 69115 Germany

**Keywords:** Patient-reported outcome measure, Measure yourself medical outcome profile, Instrument validation, Instrument translation, Sensitivity to change, Primary health care, Psychometrics, Questionnaires

## Abstract

**Background:**

Measure Yourself Medical Outcome Profile (MYMOP) is a patient-generated outcome instrument capable of measuring effects from a wide range of health care interventions. This paper reports the translation of this instrument into German (MYMOP-D) and the assessment of validity and sensitivity to change for the MYMOP-D. The instrument was piloted in a German primary care context.

**Methods:**

The translation process was conducted according to international guidelines. Recruited patients of both general practitioners and non-medical Complementary and Alternative Medicine (CAM) practitioners (“Heilpraktiker”) in the German state of Baden-Wuerttemberg completed a questionnaire comprised of the MYMOP-D and the EQ-5D. Responses were analysed to assess construct validity. For assessing the instrument’s sensitivity to change, patients received the MYMOP-D again after four weeks at which point they were also asked for their subjective views on change of symptoms. Correlation between MYMOP-D and EQ-5D and sensitivity to change as gradient in score change and as standardized response mean (SRM) were calculated.

**Results:**

476 patients from general practices and 91 patients of CAM practitioners were included. Construct validity of the MYMOP-D was given with a correlation of r = .47 with the EQ-5D. Sensitivity to change for subjective change of symptoms could only be analysed for improvement or no change of symptoms, as only 12 patients reported deterioration of symptoms. Results showed the expected smooth gradient with 2.2, 1.3, and 0.5 points of change for large, little improvement and no change, respectively. SRM for MYMOP-D Profile Score was 0.88.

**Conclusions:**

The MYMOP-D shows excellent construct validity. It is able to detect changes when symptoms in patients improve or remain unchanged. Deterioration of symptoms could not be evaluated due to too few data. With its brevity and simplicity, it might be an important tool for enhancing patient-centred care in the German health care context.

**Electronic supplementary material:**

The online version of this article (doi:10.1186/s12955-014-0112-5) contains supplementary material, which is available to authorized users.

## Background

Patient-reported outcomes (PROs) are important tools for enhancing patient-centred care. Increasingly, initiatives are being set up in different sectors of health care to drive forward the development and the use of PRO measures (PROMs). Such initiatives to date have been in different fields of medicine including oncology, surgery and rheumatology, with a focus mostly on disease-specific tools [1]-[3]. Furthermore, established standardized instruments such as the Short Form 36 (SF-36), EQ-5D or WHOQOL-BREF [4] assess general health status or quality of life. However, according to Lloyd et al., who have recently published a review on PROMs, a simple set of questions that asks patients to assess outcomes of treatment can be time and resource efficient in comparison to administering lengthy measures [5].

Although it is not a single-item outcome, a similar brief approach is pursued with the Measure Yourself Medical Outcome Profile (MYMOP) [6]. This 4-item instrument allows patients themselves to nominate up to two symptoms that are concerning them most, and to subjectively assess the change of these symptoms over time following a therapeutic intervention. The MYMOP was developed by Paterson and initially published in 1996 [6], a revised version including items on medication was validated in 1999 [7],[8]. Since then, the MYMOP has been used in several studies and has proven to be a sensitive measure of within-person change over time [8],[9]. In 2010, the MYMOP was translated to a valid and change-sensitive Chinese version [10]. Studies evaluating the MYMOP have shown its good content validity [11], feasibility [12], and sensitivity to change [13],[14]. Although MYMOP has been mostly implemented in studies in the field of Complementary and Alternative Medicine (CAM), it is capable of measuring effects from a wide variety of health care interventions and is more sensitive to change than the SF-36 instrument [6]. Due to its brevity and simplicity, it can be easily incorporated in other research settings including primary care.

The aim of this study was, therefore, to translate the MYMOP into German and pilot its use in a primary care setting. Validation of the translated version focused on construct validity and sensitivity to change.

## Methods

### Instrument and translation

The MYMOP consists of four questions. In the first two questions, patients are asked to specify up to two symptoms which have concerned them most during the last week. A third question asks for a restriction of an ADL due to the symptom(s). The fourth question focuses on general wellbeing. In an optional follow-up form, the symptoms and activities specified in the initial form are assessed again. Additionally, patients can choose to name and rate a third symptom that newly occurs and is important to them now. In terms of scoring, all questions (symptoms, activity, and general wellbeing) have to be rated on 7-point Likert-type scales with 0 as the best and 6 as the worst answer option. MYMOP Profile Scores can be calculated as a mean of the ratings. These have values between 0 and 6; the higher the score, the worse the outcome. In the initial form, up to four items are used for scoring. In the follow-up form, up to five items are used for scoring. Profile Scores of initial and follow-up forms can be compared.

The German translation was based on the version MYMOP2 (both initial and follow-up form). This is available free of charge on-line (http://www.bris.ac.uk/primaryhealthcare/resources/mymop). In keeping with established international guidelines [15], in a first step, the MYMOP was independently translated from English to German (forward translation) by two researchers with German as their mother-tongue. Both researchers agreed on a final first German version. The translated German version was given to a small patient sample (n = 16) to correct for ambiguities and to ensure practicability. Due to their feedback, the questionnaire had to be slightly adjusted. The final version was subsequently translated back into English again (reverse “back” translation) by a colleague whose mother-tongue is English and a second professional translator. The research team consented to the final reverse translation version. This version was sent to the original author of the MYMOP (C. Paterson), for final approval, which was also received.

### Validation

Objectives of the translated instrument validation process were to assess (1) construct validity and (2) sensitivity to change.

### Setting and sampling

From February 2012 to July 2013, the MYMOP-D was piloted with both patients from general practices and patients seeing independent CAM health practitioners (“Heilpraktiker”) in the German state of Baden-Württemberg. Patients > 18 years who contacted the practitioner with a newly developed symptom on a given day that consented to participate in the study were included. Patients with an insufficient command of German, middle- or higher-grade dementia patients, and patients who contacted the practitioner for a reason other than a medical consultation (i.e. follow-up prescription) were excluded from the study.

To reach a sufficient patient sample for validation and for the expected correlation (r = .30, alpha = .05, 1-beta = .20), our target sample was at least 85 patients. 779 general practitioners and 150 CAM health practitioners were invited to participate in the study. Addresses from general practices were drawn from the directory of the Association of Statutory Health Insurance Physicians (Kassenärztliche Vereinigung). Addresses for CAM health practitioners were collected from telephone books.

### Data collection

In general practices, patients were approached by a doctoral student from the Department of General Practice and Health Services Research (KK, KhH) while they were waiting for their appointment. Patients were informed about the study purpose by the doctoral student. Eligibility to participate was documented by the physicians. When filling out the questionnaire, patients could use a separate room and consider their answers undisturbed.

This procedure was not feasible for the patients of the CAM health practitioners; as the number of consulting patients per day was far less compared to that in GP practices, so that the continuous presence of the doctoral student could not be guaranteed. Therefore, we elected to train the participating CAM practitioners in informing patients on the study purpose and handing out the MYMOP-D. They were advised to approach patients consecutively and were provided with a screening list with possible reasons for excluding patients.

Participating patients noted their address on the questionnaire thus enabling a follow-up assessment after 4 weeks. Patients received the follow-up questionnaire via mail and could return it in a postage-paid envelope. Returned questionnaires were given a pseudonym number before all personal information was erased.

### Instruments for validation

To assess construct validity, a comparison with a quality-of-life questionnaire was applied. In contrast to Paterson who used the SF-36 [6], we decided to use the EQ-5D as it takes less time to complete and both measures are comparable [16]. The EQ-5D consists of 5 items with 3 statements each. Patients need to decide which of the 3 statements best fits their physical and mental state. Out of the 5 answers, an index score can be calculated [17]; the higher the score, the better the patient’s quality of life.

To assess sensitivity to change, patients had to evaluate the severity of symptoms at the 4-week follow-up compared with at the time of the initial assessment. They were asked to answer the question: “Please indicate how your symptoms have been in general during the last 4 weeks, since the initial assessment.” with the answer options being “much better”, “a little better”, “unchanged”, “a little worse”, and “much worse”. Due to organizational problems, this question was later included in follow-up questionnaires in September 2012.

### Data analysis

In a first step, patients from general practices and patients of CAM health practitioners were compared regarding their socio-demographic characteristics to facilitate a reasonable joint analysis of both groups. Described symptoms were categorized using the second edition of the International Classification of Primary Care (ICPC-2) [18]. For analysis of construct validity, MYMOP-D Profile Scores and EQ-5D scores were correlated. To confirm construct validity, a correlation of r > =.30 was expected (based on the results of Paterson [6]). Since higher scores denote worse outcome in the MYMOP-D and better outcome in the EQ-5D, the correlation was expected to be negative in tendency. For sensitivity to change, standardized response means (SRM) were calculated [6],[19]. Additionally, the mean changes in the MYMOP-D were compared among the different categories of patient-rated perceived change in severity of symptoms. A smooth gradient from deterioration to improvement is to be expected. ANOVA was used for the detection of differences in changes between groups.

For all statistical analyses, IBM SPSS version 20 was used.

The study protocol was approved by the ethics committee of the Medical Faculty of the University of Heidelberg (S-443/2011).

## Results

### Study sample

567 participants (476 patients from 34 general practices and 91 patients from 11 CAM practices) were included in our study and completed the initial questionnaire. Characteristics of the sample are to be found in Table 1.

**Table 1:** **Participant characteristics (n = 567)**

Gender n (%)	
Female	368 (64.9)
Male	199 (35.1)
Age M (SD)	50.1 (17.9) years

Due to optional questions and missing values in single questions, the sample size in the analyses varies slightly. Valid “n” are reported in the tables. Mean initial values of MYMOP-D profile score, of ratings of symptoms, activity and wellbeing, and of the EQ-5D scores are to be found in Table 2. Reported symptoms covered all chapters of the ICPC2 (except W: Pregnancy, Childbearing, Family Planning, and Y: Male Genital); most commonly reported symptoms were back pain, cough and fatigue.

**Table 2:** **MYMOP-D and EQ-5D**

	All (n = 567)	
MYMOP-D	Mean (SD)	Valid n
Symptom 1	3.9 (1.4)	565
Symptom 2	3.9 (1.3)	418
Activity	4.1 (1.5)	550
Wellbeing	3.1 (1.4)	565
Profile score	3.7 (1.1)	564
Duration of symptom 1 n (%)		566
0-4 weeks	176 (31.0)	
4-12 weeks	63 (11.1)	
3 months – 1 year	76 (13.4)	
1-5 years	103 (18.2)	
>5 years	148 (26.1)	
Medication for symptoms	318 (56.1)	567
Medication in general n (%)		564
None	105 (18.5)	
1-2	231 (40.7)	
3-4	127 (22.4)	
5-6	53 (9.3)	
7 and more	48 (8.5)	
EQ-5D M (SD)		
Health assessment on VAS	64.4 (20.3)	565
Index score	0.79 (0.23)	557

From 341 patients of the 567 patients (60.1%), we received a completed follow-up questionnaire. Patients who returned the questionnaire were more likely female (p = .02) and on average 6 years older than patients who did not fill out the follow-up form (responders M = 52.5, SD = 17.5; non-responders M = 46.3, SD = 17.8; p < .01). Initial MYMOP-D scores did not differ between responders and non-responders to follow-up, except for activity (responders M = 4.0, SD = 1.6; non-responders M = 4.3, SD = 1.4; p = .03).

### Validity

For a total of 554 patients, both MYMOP-D profile score and EQ-5D index score could be calculated for the initial completion of the questionnaire. For 13 patients, scores could not be computed due to missing values. MYMOP-D and EQ-5D correlated significantly and higher than expected with r = −.47 (p < .01).

### Sensitivity to change

On average, symptoms improved from initial to follow-up assessment based on subjective reporting via the MYMOP-D (Table 3). With the exception of the question on wellbeing, all MYMOP-D questions and the Profile Score had SRM > .80, indicating large effects of sensitivity to change [19], and wellbeing showing weak sensitivity to change with an SRM = 0.43. The EQ-5D index and the VAS in comparison showed a sensitivity to change of SRM = 0.35 and 0.17, respectively.

**Table 3:** **Mean change in MYMOP-D scores and standardized response mean (SRM)**

	Mean (SD) score at initial administration	Valid n	Mean (SD) score at follow-up	Valid n	Mean (SD) change	Valid n	SRM
MYMOP-D profile score	3.7 (1.1)	564	2.3 (1.3)	332	1.3 (1.5)	331	0.88
Symptom 1	3.9 (1.4)	565	2.4 (1.5)	337	1.5 (1.8)	337	0.80
Symptom 2	3.9 (1.3)	418	2.4 (1.5)	256	1.5 (1.7)	242	0.83
Activity	4.1 (1.5)	550	2.2 (1.8)	330	1.7 (2.0)	325	0.84
Wellbeing	3.1 (1.4)	565	2.3 (1.3)	335	0.7 (1.7)	334	0.43

**Figure 1:**
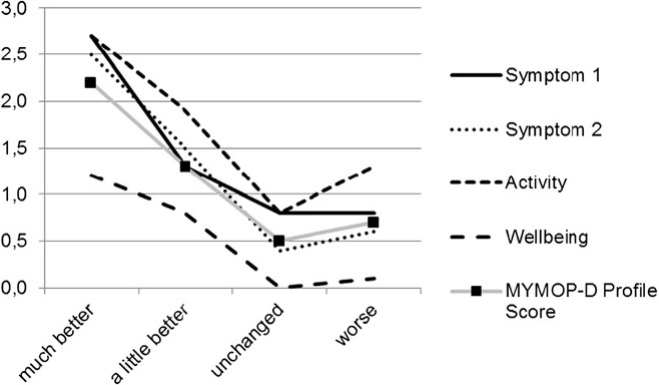
**Sensitivity to change of the MYMOP-D profile score depending on patient-rated perceived change of symptoms.**

From the total of 341 patients, who returned the follow-up questionnaire, 161 also rated the change of symptoms they perceived on the single question with answer options from “much better” to “much worse”. There were no differences in age and gender between patients who gave an overall assessment of their perceived change of symptoms and those who did not answer this question. Twelve patients (7.5%) reported deterioration of their symptoms; for 47 patients (29.2%) symptoms remained at the same level, and for 102 patients (63.4%) symptoms improved. For patients whose symptoms were subjectively “much better”, mean MYMOP-D scores on the profile score and all questions except wellbeing decreased (signifying improvement) by at least 2 points (Table 4, Figure 1). MYMOP-D scores of patients with symptoms which were subjectively “a little better” improved on average between 0.8 and 1.9 points (Table 4, Figure 1). Patients with unchanged symptoms reported mean differences between 0.0 and 0.8 points on the MYMOP-D (Table 4, Figure 1). The small sample of patients with subjectively worse symptoms consists of 10 patients reporting “a little worse” symptoms and 2 patients reporting “much worse” symptoms. Mean differences of this sample did not follow the smooth gradient; results are given in Table 4.

**Table 4:** **Sensitivity to change of the MYMOP-D profile score depending on patient-rated perceived change of symptoms**

Patient-rated perceived change of symptoms	Mean (SD) change in score (initial – follow-up)	No. of patients
Symptom 1		160
Much better^a,b,c^	2.7 (1.5)	55
A little better^d^	1.3 (1.4)	46
Unchanged^d^	0.8 (1.4)	47
Worse^d^	0.8 (1.8)	12
Symptom 2		128
Much better^a,b,c^	2.5 (1.9)	45
A little better^b,d^	1.5 (1.3)	37
Unchanged^c,d^	0.4 (1.3)	36
Worse^d^	0.6 (1.1)	10
Activity		154
Much better^b^	2.7 (2.0)	53
A little better	1.9 (1.6)	46
Unchanged^d^	0.8 (1.9)	43
Worse	1.3 (1.9)	12
Wellbeing		159
much better^b^	1.2 (1.6)	55
A little better	0.8 (1.6)	47
Unchanged^d^	0.0 (1.4)	45
Worse	0.1 (1.2)	12
MYMOP-D profile score		158
Much better^a,b,c^	2.2 (1.2)	55
A little better^b,d^	1.3 (1.2)	46
Unchanged^c,d^	0.5 (1.0)	45
Worse^d^	0.7 (1.1)	12

Significant differences (Bonferroni correction) to ^a^worse, ^b^unchanged, ^c^a little better, ^d^much better.

## Discussion

The MYMOP2 is a patient-oriented instrument; patients themselves state problem symptoms in their own words. It is applicable to primary care since the symptoms can still be classified using the ICPC2. It can also be used in CAM settings, which is of growing importance to patients (not only in Germany). The MYMOP2 has been used successfully in English-speaking countries and until now there was no validated German translation available. Based on the results of this study, the German version of the Measure Yourself Medical Outcome Profile, the MYMOP-D, proved to be both valid and sensitive to change. For construct validity, a high concordance of MYMOP-D and EQ-5D could be shown in our sample. High standardized response means after 4 weeks are reflected in a large proportion of patients who reported improved symptoms. Results also showed the expected smooth gradient in all questions and the MYMOP-D profile score for patients reporting unchanged and improved symptoms.

The correlation between MYMOP-D and EQ-5D was higher than expected on the basis of the original validation study [6]. We used the EQ-5D instead of the SF-36 that Paterson [6] and Chung et al. [10] used. Models mapping the SF-36 onto the EQ-5D showed similar results [16], so that our results are comparable to other validated versions of the MYMOP.

Sensitivity to change as measured as standardized response means is comparable to other studies using the MYMOP [6],[14]. Patients in our study reported large effects (SRM > .80) on almost all MYMOP scales, reflecting the high proportion of patients also reporting a subjective improvement of their symptoms. The EQ-5D, in comparison, was less able to detect changes in patients’ experiences of their symptoms. In a review considering different patient groups, SRMs of the EQ-5D were also weak with values not larger than .43 after 3 to 12 months [20]. It seems the EQ-5D only reacts to major impacts on quality of life.

The MYMOP-D profile score and all questions except the one regarding wellbeing were very sensitive to change. Other studies using the MYMOP also observed the question about wellbeing as the least sensitive of the questions [6],[14],[21]. Wellbeing is influenced by a multitude of factors and reported symptoms at a given point in time might be just a (minor) part of it. The MYMOP allows patients to name other factors they suspect have an influence on their health, but patients – at least in our study – rarely use this opportunity. In a clinical setting, as a tool for communication and reflecting on the therapy with the patient [6], we recommend that they should be encouraged to fill out this part of MYMOP as well. It enables medical professionals to understand patients’ underlying concepts of disease and could assist in identifying influences on wellbeing. Nevertheless, the practicability of the MYMOP-D as a communication tool in everyday general practice and with patients of CAM health practitioners remains to be demonstrated.

Baseline values and change in MYMOP-D scores are comparable to the results of other studies with patients, such as: from an acupuncture clinic [9], with rotator cuff tendinitis [22], with long-term conditions [13], or with insomnia [23]. With this background, it would be possible to conclude clinically relevant improvements from the MYMOP scores. For a clinical consultation, this could help both practitioners and patients to define appropriate therapy goals.

A review of outcome instruments for treatment of chronic low back pain advised the use of more specific instead of generic tools for measuring sensitivity to change [24]; an evaluation based on patient preference, i.e. with the MYMOP-D, is highly specific. The use of patient-preference questionnaires has several advantages: patients only rate symptoms and activities which are of immediate meaning to them, and since contents of the questionnaire are personally relevant, the problem of missing data is minimized. Improvements during therapy are easier to detect.

### Limitations of the study

Although adequate numbers of patients returned the follow-up questionnaire, only a part of this number had answered the question about the perceived change of symptoms. Too few patients reported a deterioration to be able to calculate sensitivity to change in this category. The problem with only a small proportion of patients reporting deterioration has been previously observed [9],[10] and discussed [12]. The fact is patients presenting with acute symptoms for medical advice, very rarely experience worsening symptoms four week later (and even more rarely do these patients develop chronic symptoms of disease) [12]. In our study, all patients were under current treatment for their symptom(s). Additionally, the main problem for the patient might change over time and is not considered in this questionnaire [11].

## Conclusions

The MYMOP-D proved to be a valid tool with good sensitivity to change and with excellent construct validity. For sensitivity to change over the whole spectrum, including deterioration of symptoms, more patients with deteriorating symptoms need to be assessed. Because of its brevity and simplicity, it can be easily incorporated into primary health care settings and, therefore, might be an important tool to enhance patient-centred care.

## Authors’ original submitted files for images

Below are the links to the authors’ original submitted files for images. Authors’ original file for figure 1

## Abbreviations

ANOVA: Analysis of variance
CAM: Complementary and alternative medicine
ICPC-2: International classification of primary care, second edition
M: Mean
MYMOP-D: Measure yourself medical outcome profile German version
PROs: Patient-reported outcomes
PROMs: Patient-reported outcome measures
SD: Standard deviation
SRM: Standardized response mean
