# Supplementary figures and images for: A brief patient-reported outcome instrument for primary care: German translation and validation of the Measure Yourself Medical Outcome Profile (MYMOP)
Source: Health Qual Life Outcomes. 2014 Jul 19;12(1):112. doi: 10.1186/s12955-014-0112-5 (PMC5011785; doi:10.1186/s12955-014-0112-5)

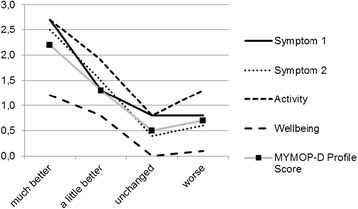

Supplement: Supplementary file 1 — Authors’ original file for figure 1 [file 12955_2014_112_MOESM1_ESM.gif]
